# Dementia in developing countries: Does education play the same role in India as in the West?

**DOI:** 10.1590/S1980-57642014DN82000008

**Published:** 2014

**Authors:** Gowri K. Iyer, Suvarna Alladi, Thomas H. Bak, Mekala Shailaja, Annapurna Mamidipudi, Amulya Rajan, Divyaraj Gollahalli, Jaydip Ray Chaudhuri, Subhash Kaul

**Affiliations:** 1 Department of Neurology, Nizam’s Institute of Medical Sciences, Hyderabad, India.; 2 Department of Psychology, Centre for Cognitive Aging and Cognitive Epidemiology (CCACE) and Centre for Clinical Brain Sciences (CCBS), University of Edinburgh.; 3 Department of Science, Technology and Society studies, Maastricht University, Netherlands.; 4 Department of Neurology, Yashoda Hospitals, Hyderabad, India.

**Keywords:** education, dementia, stroke, bilingualism, rural dwelling

## Abstract

Evidence suggests that education protects from dementia by enhancing cognitive reserve. However, this may be influenced by several socio-demographic factors. Rising numbers of dementia in India, high levels of illiteracy and heterogeneity in socio-demographic factors provide an opportunity to explore this relationship.

**Objective:**

To study the association between education and age at dementia onset, in relation to socio-demographic factors.

**Methods:**

Association between age at dementia onset and literacy was studied in relationship to potential confounding factors such as gender, bilingualism, place of dwelling, occupation, vascular risk factors, stroke, family history of dementia and dementia subtypes.

**Results:**

Case records of 648 dementia patients diagnosed in a specialist clinic in a University hospital in Hyderabad, India were examined. All patients were prospectively enrolled as part of an ongoing longitudinal project that aims to evaluate dementia subjects with detailed clinical, etiological, imaging, and follow-up studies. Of the 648 patients, 98 (15.1%) were illiterate. More than half of illiterate skilled workers were engaged in crafts and skilled agriculture unlike literates who were in trade or clerical jobs. Mean age at onset in illiterates was 60.1 years and in literates 64.5 years (p=0.0002). Factors independently associated with age at dementia onset were bilingualism, rural dwelling and stroke, but not education.

**Conclusion:**

Our study demonstrates that in India, rural dwelling, bilingualism, stroke and occupation modify the relationship between education and dementia.

## Abstract (pt)

Evidências sugerem que a educação protége de demência pelo fortalecimento da reserva cognitiva. Todavia, pode ser influenciado por vários fatores socioeconômicos. O aumento no número de demência na Índia, altos índices de analfabetismo e heterogeneidade de fatores sociodemográficos fornecem uma oportunidade para explorar estas relações.

**Objetivo:**

Estudar a associação entre educação e idade no início da demência em relação aos fatores sociodemográficos.

**Métodos:**

A associação entre idade de início da demência e alfabetismo foi estudado em relação aos potenciais fatores confundidores, como gênero, bilinguismo, local de moradia, ocupação, fatores de risco vasculares, acidente vascular cerebral (AVC), história familiar de demência e subtipos de demência.

**Resultados:**

Arquivos de 648 pacientes com demência, diagnosticados numa clínica especializada no Hospital Universitário em Hyderabad, foram avaliados. Todos os pacientes foram prospectivamente incluídos num projeto de acompanhamento longitudinal cujo objetivo é avaliar indivíduos com demência através de estudo de detalhado de acompanhamento clínico, etiológico e de imagem. Dos 648 pacientes, 98 (15%) eram analfabetos. Mais da metade dos analfabetos estavam envolvidos em trabalhos manuais ao contrário dos alfabetizados, envolvidos em comércio ou escritórios. A idade média de início em analfabetos foi de 60,1 anos e entre alfabetizados 64,5 anos de idade (p=0,0002). Os fatores independentemente associados à idade de início da demência foram bilinguismo, AVC, moradia rural, mas não educação.

**Conclusão:**

Nosso estudo demonstra que na Índia, moradia rural, bilinguismo, AVC e ocupação modificam a relação entre educação e demência.

## INTRODUCTION

Recent studies estimate that most people with dementia live in developing countries (60% in 2001, rising to 71% by 2040). This rapid increase has been attributed to the phenomenon of demographic transition, increased life expectancy, urbanization and other lifestyle factors.^1^ It is also well established that the clinical expression of dementia in any population is modifiable by several lifelong factors that enhance premorbid cognitive ability and enhance cognitive reserve.^2,3^ The main factors thought to influence cognitive reserve include education, occupation, socioeconomic environment, physical health, health behaviors, and degree of engaged lifestyle activity. Education has been most extensively studied among these and there is compelling evidence for its independent protective role.^4-14^ However, several studies report an interaction between education and other lifestyle and biological factors, specially occupation, gender, rural dwelling, cardiovascular disease and genetic factors in influencing dementia risk and its clinical expression.^15-20^ Along with the abovementioned factors, there have been some recent studies including a study from our memory clinic, that have suggested that bilingualism may delay the age at onset of dementia due to Alzheimer's disease (AD) dementia by up to five years.^21-24,63^ The potential mechanism of enhanced cognitive flexibility and control is suggested by recent studies.^25,26^ A bilingual cognitive advantage in executive functioning, has also been reported in the literature.^27-30^

India currently has the largest population of illiterate adults in the world with 287 million illiterates. While levels of education vary widely, the reported literacy rate is 74.04%, indicating that nearly a quarter of India's population continues to be illiterate.^31^ In the background of rising numbers of dementia in India, this level of illiteracy has resulted in a large burden of illiterate dementia patients. However, little is known about the profile of illiterate dementia patients in India. Further, India is unique in its heterogeneity with regard to socio-demographic factors, lifestyle practices, geographical variability, and high cardiovascular risk, but very little is known about the interaction between these factors in influencing expression of dementia. In this study, we aimed to examine the socio-demographic profile, and disease characteristics among literate and illiterate dementia patients and study the association between education, age at dementia onset and its subtypes, taking into account a range of potential confounding factors. The confounding factors include gender, bilingualism, place of dwelling, occupation, vascular risk factors, stroke, family history of dementia and dementia subtypes.

## METHODS

**Patients.** The study examined the data of 648 consecutive dementia patients diagnosed in a specialist Memory Clinic of a University hospital in Hyderabad in India between June 2006 and October 2012. All patients were participants of a longitudinal dementia registry project aimed to evaluate dementia patients with clinical, imaging and follow up studies. The patient profile is representative of the pattern seen at a tertiary, referral neurology service in an Indian city. All subjects were evaluated using a diagnostic protocol adapted from the Cambridge Memory Clinic model.^32^

The assessments were performed by trained psychologists using a structured proforma. Mini Mental State Examination (MMSE), Addenbrooke's Cognitive Examination – Revised (ACE-R) were used to assess cognition.^33^ Separate Telugu and Hindi literate and illiterate ACE-R versions were developed to account for education effects on performance on these tests, following a standard process of adaptation and validation.^34,35,37^ These were found to be useful, sensitive and specific for dementia diagnosis among literate and illiterate populations in our memory clinic setting and were used in this study.^36,37^ Clinical Dementia Rating Scale (CDR) was used to assess severity of dementia.^38^ Diagnoses of dementia and its subtypes were made based on standard criteria.^36^

The classification of patients into the following dementia types was based on standard criteria: Alzheimer's Disease (AD) was diagnosed in patients who fulfilled NINCDS-ADRDA criteria for probable and possible AD,^39^ Vascular Dementia (VaD) was diagnosed in patients who fulfilled NINDS-AIREN criteria for probable and possible VaD,^40^ mixed AD with cerebrovascular disease (CVD) was diagnosed according to NINDS-AIREN criteria,^40^ Fronto-temporal dementia (FTD) was diagnosed based on Lund-Manchester criteria,^41^ and Dementia with Lewy Bodies (DLB) was diagnosed based on the third report of the DLB Consortium.^42^

For the present study, case records of patients from the dementia registry were reviewed for these details: age of patient, sex, mono/bilingualism, age at dementia onset, occupation, rural vs. urban dwelling, family history of dementia, history of stroke, vascular risk factors and duration since the onset of first symptom of significant cognitive decline and dementia (as observed and reported by a family member).

**Education and occupation status.** Education history was obtained by interviewing a reliable family carer and based on the information obtained, patients were categorised into two groups: literates and illiterates. The operational definition for literacy was derived from the definition used by Census India 2001.43 Literates were individuals who could read and write with understanding in any language and may or may not have received any formal education. Bilinguals in this study were defined based on Mohanty's definition.^44^ National Classification of Occupations-2004, which is based on skill levels for Indian conditions, was used to classify subjects into different occupational status.^45^ Age at onset of dementia was defined as the age at which the first clinical symptom suggestive of dementia was observed by a reliable family member/carer. Informed consent was obtained from all the participants and caregivers in the study.

During the study period, of the 715 patients who were diagnosed with dementia due to various causes, 67 patients were not included in the study due to insufficient socio-demographic or clinical data , either because the family member did not provide the necessary information or patients did not complete the evaluation. The remaining 648 patients were included in the study.

The study received approval from the ethics committee of Nizam's Institute of Medical Sciences.

**Statistics.** Illiterate and literate subject groups were compared across clinical and socio-demographic factors. Comparisons between illiterate and literate groups of patients were made using independent samples t-test/one-way analysis of variance (ANOVA) followed by *post hoc* tests using Bonferroni's adjustments for continuous variables and Chi-square test for categorical variables. A univariate general linear model (GLM) was used to assess the independent association of age at onset of dementia with education and other socio-demographic and clinical variables and also to assess independent association of educational status with socio-demographic and clinical variables. Statistical analyses were performed using SPSS 20.0 for Windows software (SPSS Inc., Chicago, IL) and significance was set at p<0.05.

## RESULTS

**General characteristics of the dementia patients.** The study cohort consisted of 648 patients of which 424 (65.4%) were men, 224 (34.6%) were women. The mean age of the group at presentation was 66.2 years (range 32-92 years) and the duration of illness ranged from six months to 11 years (mean 2.3 years). AD was diagnosed in 240 (37.0%), VaD in 189 (29.2%), FTD in 116 (17.9%), DLB in 55 (8.5%), and mixed dementia in 48 (7.4%). 391 patients were bilinguals (60.3%) and 26% were from rural areas. 550 patients (84.9%) were literate and 98 (15.1%) were illiterate. Mean years of education in the literate group was 11.9 years (range 3-23 years).

**Comparison of socio-demographic features of illiterate and literate patient groups.** Illiterates were younger at presentation, were more often women and lived in rural areas. Higher proportions of illiterates were engaged in elementary occupations. There was no difference in the proportions of patients engaged in skilled occupations between the two groups (Table 1). But the profile of skilled occupations varied across the groups. 40% of illiterates were engaged in crafts like weaving, pottery and tailoring, 15% in skilled agriculture, 30% were drivers, electricians and mechanics and remaining 15% in trade, service and other occupations. Among literates, 30% were in trade, 20% were clerks, 20% were mechanics and electricians, 10% were skilled agricultural workers and 20% were service workers and in other occupations.

**Table 1:** Socio demographic profile of literate and illiterate dementia patients.

		Illiterates n=98 (15.1%)	Literates n=550 (84.9%)	p value
Age*		62.1±10.7^§^	66.9±10.7^§^	<0.0001
Gender, Men : Women^+^		45 : 53 (45.9% : 54.1%)^||^	379 : 171 (68.9% : 31.1%)^||^	<0.0001
Occupation%^¶^	Elementary^+^	7 (21.2%)^||^	6 (1.8%)^||^	0.001
	Skilled workers^+^	25 (75.8%)^||^	245 (74.0%)^||^	0.92
	Associate professionals^+^	1 (3.0%)^||^	26 (7.9%)^||^	0.55
	Professionals^+^	0 (0%)^||^	54 (16.3%)^||^	0.03
Urban^||+^		46 (51.7%)^||^	381 (78.2%)^||^	0.04
Bilinguals**^+^		18 (18.4%)^||^	373 (67.8%)^||^	<0.0001

* t-test;

+ Chi square test;

§ Standard deviation;

|| Percentage;

¶ Illiterates n=33, Literates n=331, Missing data n=147 (Housewives n=137, excluded from occupational status analysis);

** Illiterates n=46, Literates n=381, Missing data n=72.

**Education and age of onset of dementia.** Illiterate patients were found to be 4.4 years younger than literate patients at onset of dementia. The mean age at onset among illiterates was 60.1 years (SD 10.8) and among literates 64.5 years (SD 10.7) and the difference was significant (ANOVA, F1,646=13.95, p=0.0002). We explored age at onset of dementia in groups of patients with increasing years of education. While there was an increase in age at onset with increasing years of education, the association was not statistically significant (Table 2). Further, education was not found to be independently associated with age at onset of dementia after adjusting for other variables, using GLM (F1,646=0.45, p=0.83). Several lifelong experiences, demographic and biological factors are known to impact dementia risk and can influence the relationship between education and dementia. Factors that were independently found to be associated with age at onset of dementia were bilingualism (F1,646=4.89, p=0.027), rural dwelling (F1,574=22.8, p<0.0001), and stroke (F1,631=5.6, p=0.01) (Table 3).

**Table 2:** Age of onset of dementia patients across education groups.

	n	Mean, SD^+^	Illiterate vs other education groups^§^	1-7 years vs other education groups^§^	8-12 years vs other education groups^§^	13 years and above vs other education groups^§^
Illiterates	98	60.1±10.8	-	*	n.s.	**
1-7years	116	63.9±10.6	*	-	n.s.	n.s.
8-12 years	191	63.0 ± 11.2	n.s.	n.s.	-	*
13 years and above	243	66.0±10.3	**	n.s.	*	-

* p<0.05;

** p<0.001;

+ SD: Standard deviation;

§ ANOVA;

n.s. not significant.

**Table 3:** Univariate general linear model with age at onset of dementia as the dependent variable.

Factor	p value
Gender	0.98
Occupation	0.23
Education	0.83
Rural dwelling**	<0.0001
Bilingualism*	0.027
MMSE	0.72
ACE-R	0.80
Severity of dementia (CDR)	0.76
Dementia type	0.117
Family history	0.26
Stroke*	0.01
Coronary Artery Disease	0.13

MMSE: Mini Mental State Examination; ACE-R: Addenbrooke’s Cognitive Examination-Revised; CDR: Clinical Dementia Rating;

* significant at p<0.05;

** significant at p<0.01.

Additionally, in the absence of an independent association between illiteracy and age of dementia onset, we examined the possibility of occupation of illiterates contributing to this finding. The mean age at onset for the skilled illiterates was 59.2 years (n=25, SD=10.0), while the mean age at onset in illiterate subjects performing unskilled manual work was 52.8 years (n=7, SD=8.7), but the difference did not reach statistical significance (p=0.14).

We also identified factors that were independently associated with literacy, using univariate GLM. Rural dwelling (F1,574=9.3, p=0.02), occupational status (F3, 360=8.9, p<0.0001), monolingualism (F1,646=21.7, p< 0.0001), coronary artery disease (F1,633=4.0, p=0.04) and stroke (F1,631=13.4, p<0.0001) were found to be independently associated with literacy.

**Dementia characteristics in the illiterate and literate patient groups.** The duration of illness was similar between the two groups of literate and illiterate dementia patients. When illiterates reported to the clinic, they were at a more advanced stage of dementia when compared to literates. Lower scores on ACE-R and MMSE were also found in illiterates. However, when adjusted for CDR, ACE-R scores were comparable across literates and illiterates. AD dementia, FTD, and mixed dementia were equally encountered in both the groups. VaD was higher among illiterates compared to literates while DLB was found more frequently in literate group. Coronary heart disease was more common in literates while stroke was more common in illiterates (Table 4).

**Table 4:** Dementia characteristics and risk factor profile of literate and illiterate dementia patients

		Illiterates n=98 (15.1%)	Literates n=550 (84.9%)	p value
Age at presentation^§^		62.1±10.7*	66.9±10.7*	<0.0001
Age at onset^§^		60.1±10.8*	64.5±10.7*	0.0002
Duration of illness (in years)^§^		2.0±1.5*	2.3±1.8*	0.094
MMSE^§^		13.8±7.1*	18.7±7.8*	<0.0001
ACE-R^§^		42.5±23.8*	54.6±24.0*	<0.0001
CDR^||^	Mild	51 (52.0%)^+^	398 (72.4%)^+^	0.08
	Moderate	39 (39.8%)^+^	127 (23.1%)^+^	0.01
	Severe	8 (8.2%)^+^	25 (4.6%)^+^	0.24
Dementia subtype^||^	AD dementia	31 (31.6%)^+^	209 (38.0%)^+^	0.806
	FTD	18 (18.4%)^+^	98 (17.8%)^+^	0.92
	VaD	43 (43.9%)^+^	146 (26.5%)^+^	0.018
	DLB	3 (3.1%)^+^	52 (9.5%)^+^	0.08
	Mixed	3 (3.1%)^+^	45 (8.2%)^+^	0.14
Family History of dementia^||¶^		13 (14.0%)^+^	87 (16.3%)^+^	0.647
Vascular Risk Factors%^||^**	Hypertension	61 (62.2%)^+^	299 (55.7%)^+^	0.268
	Diabetes	30 (30.6%)^+^	184 (34.3%)^+^	0.561
	Smoking	18 (18.6%)^+^	66 (12.3%)^+^	0.104
	Alcoholism	9 (9.3%)^+^	71 (13.2%)^+^	0.323
	Coronary artery disease	7 (7.1%)^+^	97 (18.1%)^+^	0.007
	Stroke	38 (38.8%)^+^	123 (22.5%)^+^	0.001

MMSE: Mini Mental State Examination; ACE-R: Addenbrooke’s Cognitive Examination-Revised; CDR: Clinical Dementia Rating; AD dementia: Alzheimer Disease dementia; FTD: Frontotemporal Dementia; VaD: Vascular Dementia; DLB: Dementia with Lewy bodies;

* Standard deviation;

+ Percentage;

§ t-test;

|| Chi-square test;

¶ Illiterates n=13, Literates n=87, Missing data n=22;

** Illiterates n=98, Litertes n=535, Missing data n=15.

## DISCUSSION

Our objective in this large clinic based study of 648 dementia patients from Hyderabad was to explore the relationship between education and dementia in the Indian context. Illiterate dementia patients in our cohort had a distinctive socio-demographic profile characterised by female gender, rural dwelling and a higher frequency of monolingualism. While a significant proportion was engaged in elementary jobs, many of the illiterates were engaged in skilled occupations. They had a higher severity of dementia at initial presentation compared to literates. While literacy was associated with a delayed age at onset of dementia, after adjusting for potential confounding factors, there was no independent association between literacy and age at onset. When examining the different sub-types of dementia, the proportion of VaD was higher in the illiterates, and the proportion of DLB in the literate group. The implications of these findings will be discussed in the sections below.

In profiling the socio-demographic variables of our large dementia cohort, we found that the mean age of our dementia cohort was 66.2 years, consistent with memory clinic studies in India and other developing countries,^46-50,52^ but younger than that reported from developed countries. This is related to the socio-demographic profile of India characterised by lower life expectancy and larger proportion of patients with VaD due to a high prevalence of cardiovascular disease burden.^50-51^ Further, we included patients with FTD who are typically younger than the other dementia subtypes.^52^ 15.1% of our cohort was illiterate, compared to a much higher proportion (61.1%)^53^ in the community, suggesting that only a small proportion of illiterate dementia patients seek medical help. Illiterate patients were more often women reflecting the gender discrepancy pattern seen in the general population.^31^ Further, nearly half of the illiterate dementia patients were from rural areas, in comparison to only one-fifth in the literate group, consistent with recent census reports.^31^ The occupation profile between illiterates and literates was also different. Proportion of illiterate dementia patients employed in unskilled jobs (21%) was higher than literate patients (1.8%). The majority (75%) of patients in both literate and illiterate groups were engaged in skilled jobs, but the nature of skilled occupations differed between the two groups. Most illiterates engaged with crafts and skilled agricultural work, while literate patients were working as clerks, service workers or in sales. This profile varies from census data that reports that the largest illiterate populations in the community comprise of unskilled agricultural labourers from rural areas or casual labourers from urban areas.^53^ The subset of illiterate unskilled agricultural and casual labourers appear to have been underrepresented in our cohort, and this is most likely explained by their low socioeconomic status that is linked to low awareness about dementia and limited access to health care facilities.

The predominant finding reported in earlier studies examining the relationship between education and dementia is that education appears to protect against dementia. These results have been typically explained in the context of how early life advantages conferred by schooling contribute to cognitive reserve.^3-11,20,54^ One of the main findings of a recent meta-analysis^15^ was a strong association between low educational levels and increased risk of dementia measured by prevalence and incidence of dementia and AD dementia. In the Indian context, an epidemiological study from rural and urban areas in the state of Uttar Pradesh observed a continuous pattern of decrease in dementia prevalence with increase in educational level. The study was based on 2890 subjects aged 50 years and above, and the prevalence of dementia in the uneducated was 12.6%, which decreased to a significant level of 3.4% for subjects educated up to class 5^th^ (5 years of schooling), followed by 2.8% for classes 6^th^ to 9^th^ (6-9 years of schooling) and 2.2% for high school and onwards (12+ years of schooling).^14^ More recently, PET imaging studies demonstrated that higher education was associated with reduced glucose metabolism and higher amyloid burden in AD dementia, suggesting that the clinical expression of dementia is delayed and reduces in severity with education.^55,56^ In cross-sectional studies of Mild Cognitive Impairment (MCI) and AD dementia patients, smaller hippocampal or entorhinal cortex volumes in one study and thinner global cortical mantle in another were found amongst the more highly educated subjects in relation to less educated subjects, implying support for the cognitive reserve hypothesis.^57,58^ A recent study indicated that higher education was associated with lower hippocampal atrophy in patients with AD^59^ linking higher education levels to lower rates of incident dementia observed in epidemiological studies. Neuropathological studies have also shown that education modifies the relation of AD pathology to cognitive decline and that persons with more years of education have higher levels of cognitive function throughout adult life requiring more pathology to reach a given level of cognitive impairment.^12^

However, the nature of association between education and dementia is not that straightforward or simple. There have been studies in which the association between dementia and lower education levels has not been replicated.^15-17^ Additionally, a series of studies showed that low education increased risk of dementia only when in combination with other specific socio-demographic variables. The combination of rural residence with low education seemed to contribute to the risk of developing AD,^18^ and in a pooled analyses, it was women with lower levels of education who were at higher risk for dementia.^19^ Based on the current literature, while there appears to be a beneficial effect of education on dementia due to the building of cognitive reserve,^12,60,61^ other related factors could also possibly influence this association. In our study, we evaluated the potentially protective role of education by studying association of literacy with age at onset of dementia. In this cohort, mean age of illiterate dementia patients was 4.8 years less than educated patients (62.1 *vs* 66.9 years). However, on adjusting for potential confounding factors, education was not independently associated with age at onset of dementia. There are several possible explanations for this finding. The effect of education may be mediated by other related lifestyle and biological factors that are associated with low education.^62^ To explore this, we studied the role of a range of these factors for independent association with age at onset of dementia using GLM. Only bilingualism, stroke and rural dwelling were associated with age at dementia onset in our cohort. Bilingualism delayed age of onset, while stroke and rural dwelling advanced it. The association between bilingualism and a later onset of dementia has been previously reported.^63-66^ India is a multilingual country where each region is characterized by cultural and linguistic diversity. The reasons and opportunities for becoming a bilingual can vary from social, occupational and through formal education. In our study cohort group, we also report that the bilinguals were far more likely to be more highly educated. One possible explanation is the Indian education system which implements the "3-language formula" in the school curriculum, where the children are taught their native language or mother tongue (Telugu), the official language of the Union (English or Hindi) and a third modern Indian language or foreign language (not including the first two). Therefore, while formal education is not the only way to acquire and learn more than one language, going through formal schooling allows for more opportunities to become bilingual/multilingual."^67,68^

It is also well established that stroke and rural dwelling increase dementia risk.^14,69,70^ Since education was strongly associated with bilingualism, stroke and rural dwelling in our study, the possibility is that these factors mediated the relationship between education and dementia in our cohort set. However, given the clear evidence from epidemiological, pathological and imaging studies, that education builds cognitive reserve, it may be that while education has a protective effect, other crucial lifestyle and biological factors that follow the period of formal educational attainment may weaken or mitigate any possible protective independent effect of education on dementia.^71,72^ The lack of a differential effect of education in our cohort could also be explained by the nature of occupations that some of our illiterates were engaged in. Unlike in the general population where people with no or low education were generally manual unskilled labourers, more than half of the illiterate patients in this study were employed in occupations such as crafts and skilled agricultural work. These craft occupations are typically acquired in childhood and young adulthood in India, often as family traditions. These skills involve learning in the cognitive domains of memory, attention and visuospatial domains^73^ and this learning occurs at a time when school-going children are acquiring these cognitive abilities though formal education. It is likely that these illiterate patients have developed cognitive reserve through the process of early skill building which may be protecting them from dementia onset much like education does in the literate patients. In a supplementary analysis, done the mean age at onset of dementia in illiterate subjects working with crafts and skilled agricultural labour was about 6.4 years later than in illiterate subjects performing unskilled manual work. Though there is a trend of higher cognitive reserve in illiterates with skilled occupations compared to illiterates in unskilled occupations, it did not reach statistical significance, because of small numbers. This trend in itself is very interesting and does seem to support the fact that occupational complexity does contribute in some manner to cognitive reserve. The other protective factor in the context of our illiterate patients possibly is the support they receive from their joint families, since there is a high prevalence of joint family networks in India across all literacy levels.^74,75^ Sociological studies from India suggest that education is working against the joint family system^76^ and future studies will be required to explore the role of a joint family in affecting burden of dementia in a population with varying levels of educational status.

Our study also explored the association between literacy and dementia subtypes. AD dementia, FTD and mixed dementia were equally represented in both cohorts. However there were more patients with VaD in the illiterate group. This finding is a reflection of the strong association of stroke with illiteracy in our cohort, consistent with previous studies reporting low education as a risk factor for stroke.^77^ In contrast, the number of patients with DLB was significantly higher in the literate compared to the illiterate group (52 versus 3 patients). This finding resonates with a recent report of DLB patients having more years of education compared to AD, and greater risk of Parkinson's disease with higher education.^78^ Combined evidence of this nature seems to suggest that factors that protect in certain contexts can increase risk for disease in other contexts, but will require further exploration.

In the study, the term literacy was used to indicate an individual's ability to read and write, consistent with the common connotation of the term. However, in view of wide variability of literacy levels, the definition needs reconsideration for the term to become more pervasive and objective. Some researchers have demarcated literacy into varied components- namely prose literacy, document literacy, numeracy and problem-solving depending upon the modality of an individual's proficiency.^79^ Further, in the context of developing countries, the disadvantages of being illiterate have also been linked to the presence/absence of a literate member in the household, termed proximate or isolated illiteracy.^80^ In our study, we restricted ourselves to the broader use of the term "illiteracy' and did not formally or objectively assess level or modality of literacy. This will need to be addressed in future studies. Another limitation of the study is that a selection bias might have occurred because all the patients were those reporting at the specialist clinic and not from the community. This reflects in the smaller proportion of illiterates in the clinic cohort compared to the community, the lesser gender discrepancy (54.1% of women vs. 45.9% of men), and lower numbers of illiterates engaged in unskilled manual labour. Further, the age at onset of our cohort was nearly a decade less than reported from epidemiologic studies in India^81,82^ although consistent with Indian memory clinic studies.^83^ We also did not formally assess socioeconomic status, which is known to interact with the association between education and dementia. However, we ascertained information about patients' occupation status, which is considered to be a good indicator of socioeconomic status.

To conclude, our study demonstrates that the relationship between education and dementia can vary in different contexts and is likely to be influenced by the prevailing risk and protective factor profile of a particular community. In the context of the socio-demographic profile of a developing country like India, risk factors for dementia such as rural dwelling, low socioeconomic status, stroke and cardiovascular disease are likely to play a more crucial role than illiteracy. The stronger protective role of bilingualism in comparison to education is probably a reflection of its pervasive presence across socioeconomic strata, unlike literacy. Our finding of occupational complexity among a subset of illiterates warrants further exploration with regard to an alternative means of developing cognitive reserve. Our study therefore demonstrates that studying a population with a different pattern of variables associated with education can unearth new insights about the relationship between education and dementia. Future community-based studies combined with neuroimaging that take into account possible confounding factors are required to explore this association.

Study was conducted at Department of Neurology, Nizam's Institute of Medical Sciences, Punjagutta, Hyderabad, India - 500 082.
